# Dr. John Jordan Lenhart

**Published:** 1915-09

**Authors:** 


### OBITUARY

**Readers are requested to report promptly the death of all physicians in Western New York, or former residents of this region, or graduates of any medical school in Western New York, and to notify the families of the deceased of our desire to publish adequate obituary notices.**

**Dr. John Jordan Lenhart,** Eclectic Medical Institute of Cincinnati 1871, University of Buffalo 1878, died at his home in Bemus Point, N. Y., March 26, 1915, aged 70, of acute dilatation of the heart.

